# Systematic mutational analysis of the LytTR DNA binding domain of *Staphylococcus aureus* virulence gene transcription factor AgrA

**DOI:** 10.1093/nar/gku1015

**Published:** 2014-10-28

**Authors:** Sophie S. Nicod, Robert O. J. Weinzierl, Lynn Burchell, Andres Escalera-Maurer, Ellen H. James, Sivaramesh Wigneshweraraj

**Affiliations:** 1 MRC Centre for Molecular Microbiology and Infection, Imperial College London, London, UK; 2 Department of Life Sciences, Imperial College London, London, UK

## Abstract

Most DNA-binding bacterial transcription factors contact DNA through a recognition α-helix in their DNA-binding domains. An emerging class of DNA-binding transcription factors, predominantly found in pathogenic bacteria interact with the DNA via a relatively novel type of DNA-binding domain, called the LytTR domain, which mainly comprises β strands. Even though the crystal structure of the LytTR domain of the virulence gene transcription factor AgrA from *Staphylococcus aureus* bound to its cognate DNA sequence is available, the contribution of specific amino acid residues in the LytTR domain of AgrA to transcription activation remains elusive. Here, for the first time, we have systematically investigated the role of amino acid residues in transcription activation in a LytTR domain-containing transcription factor. Our analysis, which involves *in vivo* and *in vitro* analyses and molecular dynamics simulations of *S. aureus* AgrA identifies a highly conserved tyrosine residue, Y229, as a major amino acid determinant for maximal activation of transcription by AgrA and provides novel insights into structure–function relationships in *S. aureus* AgrA.

## INTRODUCTION

Bacteria predominantly rely on two-component signal transduction systems (TCS) to sense and adapt gene expression patterns to constantly changing environments. The typical bacterial TCS comprises signal input and response output components, typically represented by a histidine kinase (HK) and a response regulator (RR), respectively. In response to a signal, the HK becomes phosphorylated and subsequently transphosphorylates its cognate RR, thereby activating the RR to elicit the output response. Most RRs contain two domains: a conserved amino-terminal regulatory domain and a variable carboxyl-terminal effector domain. The majority of RRs are transcription factors (hereafter referred to as RR-TF) with their carboxyl-terminal domain containing a DNA-binding motif, which allows recognition of tandem or inverted repeating DNA elements located upstream of promoters of genes and determinants for interaction with the RNA polymerase for modulating the transcriptional response. The carboxyl-terminal domain of the majority of RR-TFs contains DNA-binding motifs that belong to extensively characterized structural families and include the winged-helix motif of the OmpR/PhoB family, helix-turn-helix motif of the NtrC family and four-helix-helix-turn-helix of the NarL/FixJ family (1,2). In contrast, a small number of RR-TFs, mostly in γ-proteobacteria and firmicutes, have an unusual and relatively poorly characterized carboxyl-terminal domain, called the LytTR domain, which predominantly comprises β strands (3,4). Intriguingly, LytTR domain containing RR-TFs are disproportionately involved in the regulation of virulence gene expression. Hence, the role of LytTR domain containing RR-TFs has been widely studied in many bacterial pathogens (5–7). The molecular interactions between the LytTR domain and DNA have been recently elucidated from the crystal structure of the DNA-bound complex of the LytTR domain of *Staphylococcus aureus* accessory gene regulator A (AgrA), which is a cell-density (quorum) responsive global virulence-associated RR-TF (4,8).

**Figure 1.**
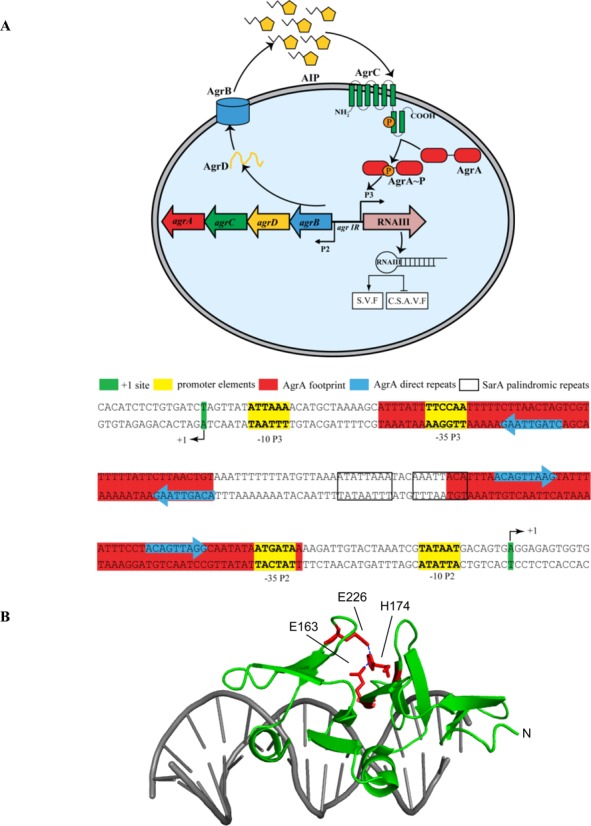

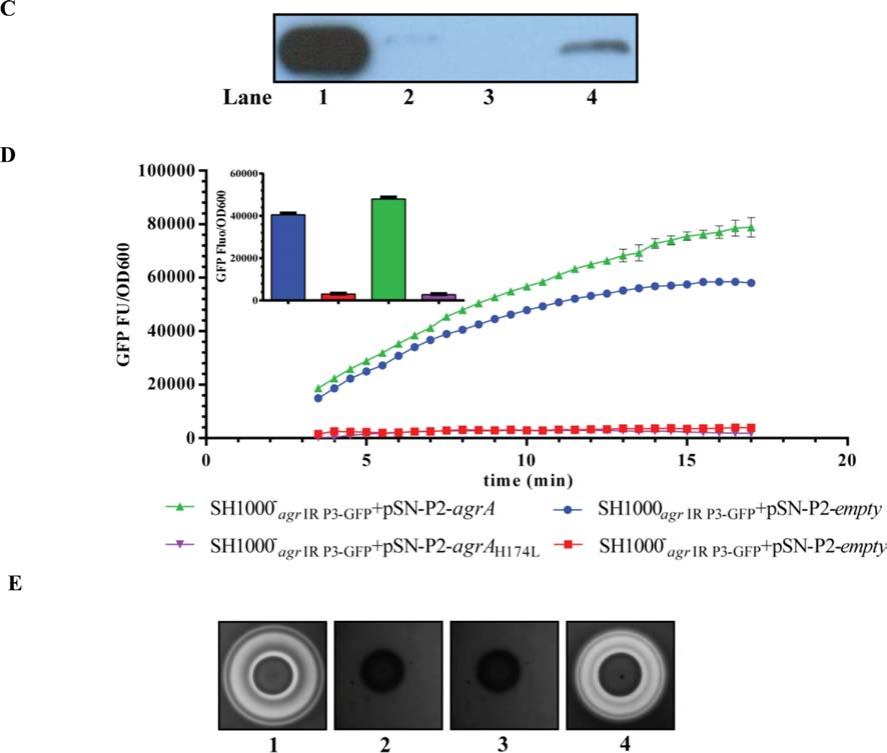
Establishing an experimental system to assess AgrA activity in *Staphylococcus aureus*. (**A**) Schematic representation of the *agr* operon organization and regulation in *S. aureus*. Secreted virulence factors (SVF) and cell-surface-associated virulence factors (CSAVF). The nucleotide sequence of the *S. aureus agr* operon intergenic region with the different regions of relevance to this study indicated. (**B**) Ribbon representation of the AgrA LytTR domain–DNA complex crystal structure (the LytTR domain and DNA are colored in green and gray, respectively). H174, E163 and E226 are highlighted in red and the salt bridge interactions indicated by dotted lines. (**C**) Western blot indicating AgrA protein level in whole cell lysate of *S. aureus* SH1000^−^ + pSN-P2-*agrA* (lane 1), SH1000^−^ + pSN-P2-*empty* (lane 2), SH1001 + pSN-P2-*empty* (lane 3) and SH1000 + pSN-P2-*empty* (lane 4) strains grown overnight in TSB. (**D**) Graph showing GFP expression [as GFP fluorescence units (GFP-FU)] as a function of growth (OD600) over time for *S. aureus* SH1000^−^_agr IR P3-GFP_ + pSN-P2-*agrA* (green), SH1000^−^_agr IR P3-GFP_ + pSN-P2-*empty* (red), SH1000^−^_agr IR P3-GFP_ + pSN-P2-*agrA*_H174L_ (purple) and SH1000 _agr IR P3-GFP_ + pSN-P2-*empty* (blue) strains grown in TSB. The bar chart in the insert represents the GFP expression of the same samples (color coded accordingly) at the 8 h time point. (**E**) Sheep blood agar hemolysis assay with SH1000^−^_agr IR P3-GFP_ + pSN-P2-*agrA* (panel 1), SH1000^−^_agr IR P3-GFP_ + pSN-P2-*agrA*_H174L_ (panel 2), SH1000^−^_agr IR P3-GFP_ + pSN-P2-*empty* (panel 3) and SH1000_agr IR P3-GFP_ + pSN-P2-*empty* (panel 4). Data for (C-E) were obtained from at least three biological replicates and for (C) and (E) representative results are shown.

The quorum-dependent regulation of AgrA activity in *S. aureus* is well understood (Figure 1A): the binding of the quorum signal (the autoinducing peptide, a peptide thiolactone, product of the *agrD* gene; (8)) to the HK AgrC results in the autophosphorylation of AgrC. Phosphorylation of AgrA by AgrC ‘activates’ AgrA thus allowing AgrA to bind to specific sites in the intergenic region of the *agr* operon and activate transcription from divergent promoters, P2 and P3. The P2 transcript contains the *agrBDCA* genes; the P3 transcript (RNAIII) is the pleiotropic effector molecule of the *agr* response (8). RNAIII is directly responsible for post-transcriptional regulation of multiple virulence factors such as α- and δ-hemolysin (which is encoded within the RNAIII transcript) (8,9). AgrA also directly activates transcription of genes *psm*α, *psm*β and *psm-mec* that encode phenol-soluble modulins (PSMs) (which play a key role in immune evasion by *S. aureus* owing to their leukocidal activity) (10). Furthermore, the LytTR domain of AgrA also serves as a redox sensor and controls the expression of the *bsaA* gene that encodes the glutathione peroxidase, which allows bacterial cells to cope with oxidative stress (11). In this scenario, oxidative stress results in the formation of an intramolecular disulfide bond between aa C199 and C228 in the LytTR domain, which causes the dissociation of AgrA from its cognate DNA sequence located immediately downstream of the *bsaA* promoter and thereby leading to the derepression of *bsaA* transcription. AgrA binds as a dimer to two direct repeats located in the intergenic region of the *agr* operon immediately upstream of the P2 and P3 promoters, with the center of the promoter proximal direct repeats located approximately at position -60 with respect to the transcription start sites at +1 for P2 and P3 (Figure 1A) (12). The *agr* operon intergenic region also contains binding sites for several other global TFs, notably SarA, which binds as a dimer to sites located between the direct repeats bound by AgrA (Figure 1A) (13). It seems that SarA and AgrA co-activate transcription from the P2 promoter (14,15). Therefore, based on the proximity of both AgrA-binding sites to the P2 and P3 promoters, respectively, it is possible that transcription activation at P2 and P3 involves protein–protein interaction between AgrA, SarA and the RNA polymerase (RNAp). Further, the -35 and -10 consensus promoter elements of all known AgrA-activated promoters in *S. aureus* have a suboptimal spacer length. For instance, the -35 and -10 consensus promoter elements of the P2 and P3 promoters of the *agr* operon are separated by 18 and 20 nucleotides, respectively, instead of the optimal 17 nucleotides long spacer region found at most promoters. Previous studies have suggested that transcription activation by AgrA involves a DNA-bending step (14). Such a mechanism could compensate for the suboptimal spacer length at the P2 and P3 promoters. Consistent with this view, shortening of the P2 and P3 promoter spacer region to the optimal 17 nucleotides length leads to increased AgrA-independent transcription from both promoters (16).

To date, only amino acid (aa) residues required for binding of LytTR domain containing TFs to DNA have been identified (4,5), but the aa residues in the LytTR domain involved in transcription activation are not known. In this study, we strategically targeted 74 of 101 aa residues in the LytTR domain (aa residues 137–238) of *S. aureus* AgrA for substitution with alanine and studied their contribution to transcription activation at the *agr* operon *in vivo* and *in vitro*. Our results, which represent the first comprehensive mutational analysis of an LytTR domain containing TF, are discussed in the context of structure–function relationships and biology of the *S. aureus* AgrA.

## MATERIALS AND METHODS

### Bacterial strains, plasmids and DNA manipulation

The bacterial strains and plasmids used in this study are listed in Supplementary Table S1 and sequences of primers used for DNA manipulation and cloning are listed in Supplementary Table S2. *Escherichia coli* and *S. aureus* were grown in Luria broth (LB) and tryptic soy broth (TSB), respectively. Where appropriate, antibiotics were added to the growth media at the following concentrations: ampicillin, 100 μg/ml (*E. coli*); kanamycin, 90 μg/ml (*S. aureus*), 50 μg/ml (*E. coli*) and chloramphenicol, 7.5 μg/ml (*S. aureus*). Anhydrotetracycline was added at a concentration of 200 ng/ml where appropriate. *Escherichia coli* XL1-blue, DC10B and BL21 (DE3) cells were transformed with plasmid DNA using the standard heat-shock transformation protocol. *Staphylococcus aureus* RN4220, SH1000, SH1000^−^ and SH1001 cells were transformed by electroporation with a Gene Pulser (Bio-Rad) with settings: 2.5 kV, 0.25 μFD, 100 Ω in a 1 mm cuvette. Following this, cells were recovered in brain-heart infusion (BHI) supplemented with 0.5 M sucrose for 1 h before being plated onto the appropriate selection plates. Transduction into target strains of *S. aureus* was carried out with φ11 following published protocols (17). All restriction enzymes and DNA modification enzymes were purchased from New England BioLabs. Plasmids and genomic DNA were extracted with the Qiagen plasmid miniprep kit and the Wizard bacterial genomic DNA kit (Promega), respectively, according to the manufacturers’ instructions. The SH1000^−^_agr IR P3-GFP_ strain was constructed exactly as previously described by phage transduction of the reporter construct from SH1000_agr IR P3-GFP_ (18). Plasmid pSN-P2-*agrA* was constructed by PCR amplifying the P2 promoter region from the pCL55*_agr_*_IR P2-GFP_ (18) plasmid containing a region at the 3′ end homologous to the 5′ end of the *agrA* gene of *S. aureus* SH1000 strain and containing the RBS using primers P2-RBS F and P2-RBS R. The *agrA* gene was amplified from the chromosome of *S. aureus* SH1000 using primers P2-AgrA F and P2-AgrA R. The transcription terminator was amplified from the pCN44 (19) plasmid containing a region at the 5′ end homologous to the 3′ end of *agrA* using primers P2-TT F and P2-TT R. The three fragments were fused together by PCR using primers P2-RBS F, P2-RBS R and P2-TT R. The 1226 bp long *BamH*I and *Kpn*I digested PCR fragment was then ligated into the *BamH*I and *Kpn*I sites of pCN34 (19) plasmid. Plasmid pSN-*itet-agrA* was constructed by PCR amplification of the *agrA* gene and terminator region from the pSN-P2-*agrA* plasmid using primers AgrA pCN34itet F containing the RBS and AgrA pCN34itet R. The 1059 bp long fragment was digested with *Kpn*I and *EcoR*I and was then ligated into the *Kpn*I and *EcoR*I sites of the pCN34*itet* plasmid (20). Plasmid pSN-agrA was constructed by PCR amplifying *agrA* from the chromosome of *S. aureus* SH1000 using primers AgrA pTYB2 F and AgrA pTYB2 F and ligating the 731 bp long*Nde*I and *Sma*I digested PCR fragment into the *Nde*I and *Sma*I sites of pTYB2.

### Purification of proteins

*Staphylococcus aureus* core RNAp and σ^A^ (from pJR28-[6His]*rpoD*) were prepared exactly as previously described by Reynolds & Wigneshweraraj (16). Recombinant AgrA was purified as follows: *Escherichia coli* strain ER2566 containing pSN-agrA was grown at 37°C. At OD_600 nm_ ∼0.6, the cells were temperature shifted to 16°C for 30 min, and the expression of AgrA was induced with 0.25 mM IPTG. The cells were harvested after 17 h at 16°C. AgrA was purified using the IMPACT^TM^ kit (New England Biolabs) according to the manufacturer's instructions. Briefly, the cells were lysed in a buffer containing 20 mM Tris-HCl (pH9), 1 M NaCl and 1 mM EDTA (column buffer) and centrifuged to remove cellular debris. The supernatant was then loaded on a 10 ml gravity flow column (Bio-Rad) packed with 2 ml Chitin Resin (New England Biolabs). The column was washed with 20 bed volumes of column buffer and the protein was cleaved from the intein tag after incubation for 16 h at 4°C in three bed volumes of cleavage buffer [column buffer + 200 mM DTT]. The protein was concentrated using Amicon Ultracel-10K (Millipore) and dialysed in a storage buffer [10 mM Tris-HCl (pH8), 50 mM NaCl, 0.1 mM EDTA, 1 mM DTT and 20% (vol/vol) glycerol].

### Bacterial growth and GFP expression assays

These were conducted exactly as described previously (18). Briefly, simultaneous growth (OD_600 nm_) and GFP fluorescence measurements (with excitation and emission filters of 485 and 520 nm, respectively) were performed in 96-well black microtiter plates with clear bottoms (Corning) in a POLARstar Omega multiwell plate reader (BMG Labtech). At least three biological replicates (each defined as a single colony) were performed for each growth curve.

### Blood agar hemolysis assay

Bacteria were grown for 16 h in TSB culture, then 20 μl aliquots were plated onto Columbia Agar containing 5% sheep's blood and left to grow for 16 h at 37°C and then 24 h at 4°C prior to image capture of the plates.

### Western blotting for AgrA

Western blotting was performed using polyclonal antibodies against AgrA (raised in rabbits against recombinant AgrA by Eurogentec (used at 1:250 dilution) and anti-rabbit-horseradish peroxidase-conjugated antibodies (Dako; used at 1:3000 dilution) as primary and secondary antibodies, respectively, following standard laboratory protocols and as described by James *et al*. (18).

### Electrophoretic gel mobility shift assays (EMSA)

Phosphorylation of AgrA was carried out by pre-incubating 3 μM AgrA with 50 mM acetyl phosphate and 5 mM MgCl_2_ for 1 h at 37°C. Ten microliter binding reactions were set up in a reaction buffer [10 mM HEPES (pH 7.6), 50 mM KCl, 1 mM EDTA, 2 mM DTT, 0.5% (v/v) TritonX-100 and 5% (v/v) glycerol] using 1 μM final concentration of phosphorylated AgrA and 10 nM of a 214 bp long γ-32P-labeled DNA fragment representing the *agr* operon intergenic region including the P2 and P3 core promoter sequences up to position +11 of the P2 promoter and +15 of the P3 promoter. The DNA probe for the EMSA was prepared by PCR amplification of the *agr* operon intergenic region from the *S. aureus* SH1000 chromosome using primers P2-IR-P3 F and P2-IR-P3 R. The phosphorylated AgrA was incubated in reaction buffer for 10 min at 37°C before adding the DNA probe. The reactions were incubated at 37°C for another 10 min and stopped with native gel loading dye [reaction buffer + 50% (v/v) glycerol and 0.05% (w/v) bromophenol blue] and resolved on a 4–20% (w/v) gradient native polyacrylamide gel. The dried gel was visualized and quantified using a GE Typhoon FLA 2000 PhosphorImager and the Image Quant TL software, respectively.

### *In vitro* transcription assays

The binding reactions for the *in vitro* transcription assays were conducted as described above; however, pJR_P2+P3_ was used as promoter template in transcription buffer [40 mM Tris–acetate (pH 7.9), 100 mM NaCl, 20 mM MgCl_2_ and 0.2 mM DTT]. The *in vitro* transcription assay was conducted as described by Reynolds & Wigneshweraraj (16). Briefly, 50 nM *S. aureus* core RNAp and 200 nM *S. aureus* σ^A^, 1 μM AgrA (phosphorylated as described above) and 10 nM pJR_P2+P3_ were incubated at 37°C for 5 min separately and then mixed and incubated for another 5 min. RNA synthesis was initiated by adding an elongation mix containing 0.5 mM ATP, CTP and GTP; 0.25 mM UTP; 0.75 μCi of α-^32^P UTP and the reaction was incubated for a further 10 min at 37°C. The reactions were stopped with stop dye [3% (w/v) xylene cyanol, 3% (w/vl) bromophenol blue and 20 mM EDTA in deionized formamide)] and resolved on a 10% (w/v) urea-denaturing polyacrylamide gel. The dried gel was visualized and quantified using a GE Typhoon FLA 2000 PhosphorImager and the Image Quant TL software, respectively.

### DNA-bending assays

The DNA-bending assays were conducted exactly as described by Reyes *et al*. (14). Plasmid pAM1847 was digested with *EcoR*I, *Hind*III, *BstN*I, *EcoR*V, *Nhe*I and *BamH*I. The fragments were purified using the Qiagen PCR purification kit and individually labeled with γ-^32^P ATP. Seven nanograms of the labeled fragments were then incubated with 1 μM of phosphorylated AgrA in a reaction buffer with 0.3 μg of sonicated calf thymus at room temperature for 20 min. The reactions were stopped with native loading dye and resolved on a 4.5% (w/v) native polyacrylamide gel. The dried gel was visualized and quantified using a GE Typhoon FLA 2000 PhosphorImager and the Image Quant TL software, respectively.

### Molecular dynamics (MD) simulations

The protonation states of residues present in the LytTR DNA-binding domain in PDB4G4K (4) were computed using H++ (21). The resulting atomic coordinates were embedded in a TIP3P (22) cubic water box extending for a minimum of 15 Å beyond the protein structure. The assembly was charge-neutralized, adjusted to 150 mM NaCl and parameterized with the AMBER99SB (23) force field. Simulations were run on Nvidia GPUs certified to carry out accurately reproducible calculations using the AMBER 12 package (24). The parameterized structures were energetically minimized by rapid descent and repeated annealing minimization before equilibration at 300^o^Kelvin and one atmosphere of pressure (NPT ensemble). For all MD simulations, the bond length between hydrogen and heavy atoms was fixed using the SHAKE algorithm (25). The time-step was 2 femtoseconds and the Particle Mesh Ewald (26) cut-off distance was set to 8 Å. The simulation engine was pmemd.cuda, a CUDA-accelerated MD production engine based on a hybrid single-/double precision (SPFP) algorithm. Conventional MD simulations were initially carried out for 50 ns to obtain representative values for the total potential and dihedral energy values for adjustment in the subsequent dual-boost accelerated MD (aMD) production runs. For boosting the dihedral potential, an energy contribution of 3.5 kcal/mol/residue per degree of freedom was assumed. A boost factor (α) of 0.2 was used for adjustment of the dihedral and potential energy in all aMD simulations (the complete data sets are available from one of the authors [ROJW] upon request). All aMD simulations lasted 100 ns, but are likely due to the acceleration to represent protein motions occurring in the tens to hundreds of microseconds range in real time (27). Trajectories were processed, visualized and analyzed using the Visual Molecular Dynamics (VMD) suite (28).

## RESULTS

### Establishing an experimental system to asses AgrA activity in *S. aureus*

Mutant *S. aureus* strain SH1000^−^ displays an *agr*-defective phenotype and previous work by Tsompanidou *et al*. showed that wild-type level of *agr* activity can be restored in SH1000^−^ in the presence of plasmid-borne AgrA (29). We obtained the nucleotide sequence of *agrA* from SH1000^−^ and identified that it contains a leucine substitution at a conserved histidine at aa position 174 (H174) in the LytTR domain (Supplementary Figure S1). In the crystal structure of the AgrA LytTR domain, aa H174 forms a salt bridge with aa E163 and E226, which contributes to stabilizing the interface between three β sheets in the LytTR domain (Figure 1B) (4). Therefore, it is likely that a leucine substitution at aa H174L compromises the overall structural integrity of AgrA, rendering it unavailable for transcription activation. As expected, AgrA protein was detectable in whole-cell lysates of *agr*-positive SH1000 (the isogenic parent of SH1000^−^), but was hardly detectable in whole-cell extracts of SH1000^−^ on western blots using polyclonal anti-AgrA antibodies (Figure 1C). Since the nucleotide sequence upstream of *agrA* including intergenic region and the P2 and P3 promoter seqences were intact in SH1000^−^, these results are consistent with the view that the H174L mutation destablizes the structural integrity of AgrA in SH1000^−^ and thereby makes it unavailable to activate transcription from P2 and P3 promoters of the *agr* operon. To ascertain that this is indeed the case, we placed a transcriptional fusion of P3 to GFP at the *geh* locus on the SH1000^−^ chromosome (creating SH1000^−^_agr IR P3-GFP_) and measured GFP fluorescence (indicating transcription from the P3 promoter) as a function of cell growth in the presence of plasmid-borne wild-type AgrA (pSN-P2-*agrA*) (Supplementary Table S1). In pSN-P2-*agrA*, the transcription of *agrA* is driven from its native promoter P2 and is dependent on functional AgrA. In other words, the transcription of *agrA* from pSN-P2-*agrA* will be autocatalytic as it is the case at the P2 promoter at the native *agr* locus. As shown in Figure 1D and as expected, P3 activity was not detected in SH1000^−^_agr IR P3-GFP_ containing pSN-P2-*empty* or pSN-P2-*agrA*_H174L_. However, P3 activity was restored in SH1000^−^_agr IR P3-GFP_ containing the pSN-P2-*agrA*. Further, since *agr* dysfunction is associated with reduced β-hemolytic activity, we used a blood agar plate hemolysis assay to confirm that β-hemolytic activity is restored in SH1000^−^_agr IR P3-GFP_ containing pSN-P2-*agrA*, but not in the SH1000^−^_agr IR P3-GFP_ containing pSN-P2-*agrA*_H174L_ or pSN-P2-*empty* (Figure 1E). We note a large difference seen in AgrA levels between in the *agr*-positive SH1000_agr IR P3-GFP_ cells (Figure 1C, lane 4) and the complemented *agrA*-defective SH1000^−^_agr IR P3-GFP_ + pSN-P2-*agrA* cells (Figure 1C, lane 1). This is as expected since there are at least 25–30 additional copies (which equates to the copy number of pSN-P2-*agrA*) of *agrA* in SH1000^−^_agr IR P3-GFP_ + pSN-P2-*agrA* than in SH1000_agr IR P3-GFP_, which will only contain one copy of *agrA*. However, rate-limiting factor for AgrA activation is AgrC, which will be present at wild-type levels in the SH1000^−^_agr IR P3-GFP_ + pSN-P2-*agrA* cells, it is unlikely that excess AgrA in SH1000^−^_agr IR P3-GFP_ + pSN-P2-*agrA* cells will have any detectable biological impact on AgrA function as it is evident in results shown in Figure 1D and E. In summary, the results unambigiously establish that SH1000^−^_agr IR P3-GFP_ in combination with plasmid-derived AgrA (from pSN-P2-*agrA*) can be used as a reporter strain to accurately measure AgrA activity in *S. aureus*.

### Alanine-scanning mutagenesis analysis of AgrA identifies four amino acid residues potentially important for transcription activation in the LytTR domain

**Figure 2.**
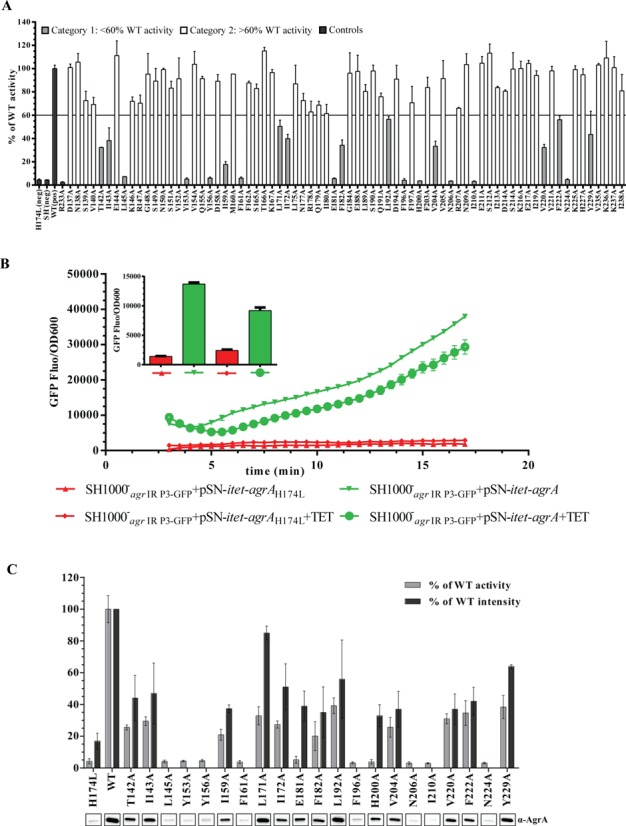

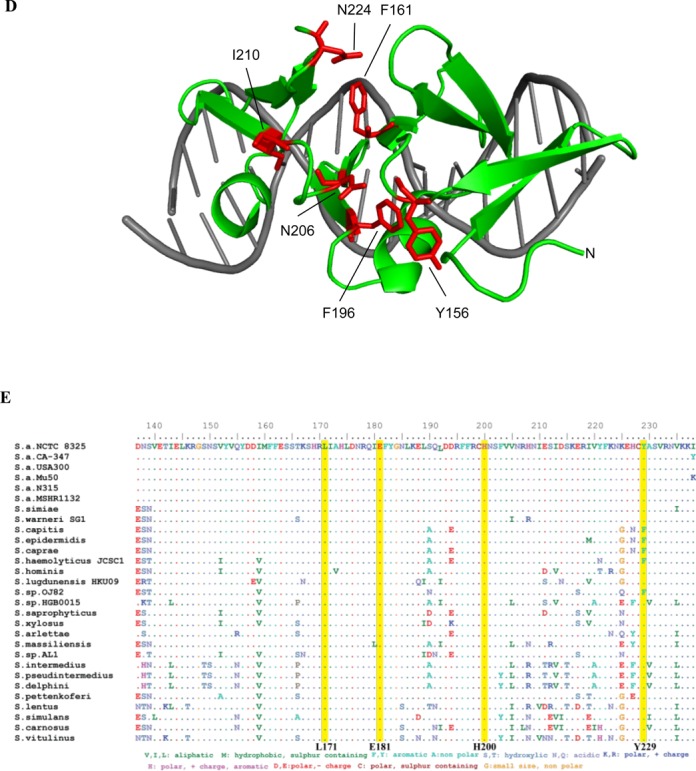
Systematic mutational analysis of *Staphylococcus aureus* AgrA LytTR domain. (**A**) Graph showing GFP expression [as GFP fluorescence units (GFP-FU)] as a function of growth (OD600) relative to the SH1000^−^_agr IR P3-GFP_ + pSN-P2-*agrA* (WT) for each single alanine mutant after 8 h of growth in TSB. AgrA mutants displaying more than 60% activity compared to the wild-type AgrA are shown in white, the AgrA mutants displaying less than 60% activity compared to the wild-type AgrA are shown in grey and the controls (SH1000^−^_agr IR P3-GFP_ + pSN-P2-*agrA*_H174L_, SH1000^−^_agr IR P3-GFP_ + pSN-P2-*empty*, SH1000^−^_agr IR P3-GFP_ + pSN-P2-*agrA* and SH1000^−^_agr IR P3-GFP_ + pSN-P2-*agrA*_R233A_) are shown in black. (**B**) Graphs showing GFP expression [as GFP fluorescence units (GFP-FU)] as a function of growth (OD600) over time for SH1000^−^_agr IR P3-GFP_ + pSN-*itet*-*agrA* (green lines) and SH1000^−^_agr IR P3-GFP_ + pSN-*itet*-*agrA*_H174L_ (red lines) strains grown in TSB with and without anhydrotetracycline. The bar chart in the insert represents the GFP expression of the same samples (color coded accordingly) at the 8 h time point. (**C**) Graph showing GFP expression [as GFP fluorescence units (GFP-FU)] as a function of growth (OD600) relative to the SH1000^−^_agr IR P3-GFP_ + pSN-*itet*-*agrA* (WT) for each of the 21 single alanine mutant displaying less than 60% wild-type activity after 8 h of growth in TSB. A section of the western blot image indicating AgrA detected in whole cell lysates for each mutant is shown under the graph. For the 13 mutants that are detectably expressed, the quantification of the intensity of the band corresponding to AgrA mutants relative to the intensity of the band corresponding to the wild-type AgrA level is shown on the graph. Data for (A–C) were obtained from at least three biological replicates. **(D)** As in Figure 1B with the six of the eight aa residues (Y156, F161, F196, N206, I210 and N224), which when changed to alanine appear to impair the gross structural stability of AgrA indicated in red. **(E)** Multiple sequence alignment of the AgrA LytTR domain of representative staphylococci strains. Conserved residues are represented by a dot. The aa residues displaying similar colors have similar properties. The putative TAD mutants are highlighted in yellow.

To indentify aa residues in the LytTR domain involved in transcription activation, we generated a mutant library of AgrA by alanine-scanning mutagenesis of the LytTR domain using pSN-P2-*agrA* as the template. Based on the crystal structure of the AgrA LytTR domain–DNA complex (4), we only targeted residues for alanine substitution that were neither implicated in DNA binding, nor known to be important for maintaining the structrual fold of the LytTR domain. We also constructed the previously described DNA-binding defective mutant AgrA_R233A_ to use as a negative control. SH1000^−^_agr IR P3-GFP_ cells were transformed with the library comprising a total of 74 AgrA mutants in pSN-P2-*agrA* and mutant AgrA activity was determined by measuring GFP fluorescence as a function of cell density (OD_600 nm_) after 8 h of growth in rich media. As expected, GFP activity was bareley detected in SH1000^−^_agr IR P3-GFP_ containing pSN-P2-*agrA*_R233A_ and pSN-P2-*agrA*_H174L_ in comparison to SH1000^−^_agr IR P3-GFP_ containing pSN-P2-*agrA* (Figure 2A). Based on the spread of the activities of the mutant AgrA library in SH1000^−^_agr IR P3-GFP_, the AgrA mutants were categorized into two activity groups: <60% (21 mutants) and >60% (51 mutants) activity relative to wild-type AgrA (Figure 2A). Mutants in the first group, which hereafter are referred to as putative transcription-activation-defective (TAD) mutants, were selected for further analysis.

To establish that the TAD property is not caused by the alanine substitution adversely affecting the stability of the mutant AgrA protein under the assay conditions (after 8 h of growth in rich media), we transferred the 21 putative TAD mutations into plasmid pSN-*itet*-*agrA*, so that expression of AgrA can be induced with anhydrous tetracycline and thus independent of the P2 promoter (see above). In other words, unlike with pSN-P2-*agrA*, transcription of *agrA* from pSN-*itet*-*agrA* will not be autocatalytic and therefore will be independent of AgrA. This approach will avoid complications in the interpretation of the results arising from a putative TAD mutant failing to or poorly activating its own transcription. Initially, we tested if P3 activity in SH1000^−^_agr IR P3-GFP_ can be restored with pSN-*itet*-*agrA* in the presence of anhydrotetracycline. As shown in Figure 2B, P3 activity was restored to comparable levels in SH1000^−^_agr IR P3-GFP_ containing pSN-*itet*-*agrA*, both in the presence and absence of anhydrotetracycline, but, as expected, not in SH1000^−^_agr IR P3-GFP_ containing pSN-*itet*-*agrA*_H174L_ even in the presence of anhydrotetracycline. It seems that leaky expression of AgrA from pSN-*itet*-*agrA* is sufficient to restore full P3 activity in SH1000^−^_agr IR P3-GFP_ containing pSN-*itet*-*agrA*, therefore all downstream experiments involving pSN-*itet*-*agrA* were conducted in the absence of anhydrotetracycline. We next monitored, by Western blotting using anti-AgrA antibodies, the accumulation of AgrA in SH1000^−^_agr IR P3-GFP_ + pSN-*itet*-*agrA* (in the absence of anhydrotetracycline) over time to determine a time point at which saturation in AgrA levels has occurred. As shown in Supplementary Figure S2, AgrA levels begun to saturate approximately after 6 h of growth in rich media. Therefore, we used the 8 h post-inoculation time point to compare the amount of AgrA in the cell and GFP fluorescence (indicative of P3 activity) in SH1000^−^_agr IR P3-GFP_ cells producing the TAD mutants from pSN-*itet*-*agrA* with SH1000^−^_agr IR P3-GFP_ + pSN-*itet*-*agrA* expressing wild-type AgrA. As shown in Figure 2C, 8 of the 21 mutants are not detectably expressed, which suggests that alanine substitution at positions L145, Y153, Y156, F161, F196, N206, I210 and N224 in the LytTR domain of AgrA could impair the gross structural stability of AgrA under the assay conditions. Consistent with this view, side-chains of Y156, F161, F196, N206, I210 and N224 are either fully or partly buried within the hydrophobic core of AgrA (Figure 2D). The remaining 13 AgrA mutants are detectably expressed at varying levels compared to wild-type AgrA under the assay conditions. Notably, even though L171A, E181A, H200A and Y229A mutants are expressed to levels at least ≥ 35–50% of that of wild-type AgrA, a relatively reduced level of P3 activity (by ≥60% reduction in activity relative to wild-type AgrA) is seen in cells containing these mutants. Thus, the results suggest that aa residues L171, E181, H200 and Y229 in the LytTR domain could potentially play a significant role in transcription activation by AgrA. This conclusion is further substantiated by the 100% identity of L171, E181 and H200 among *agrA* alleles in representative staphylococcal species; Y229 is also highly conserved, being replaced by the similar phenylalanine in 5 of the 29 sequences (Figure 2E).

### Conserved amino acid Y229 in the LytTR domain of AgrA is not required for DNA binding or bending but for transcription activation

**Figure 3.**
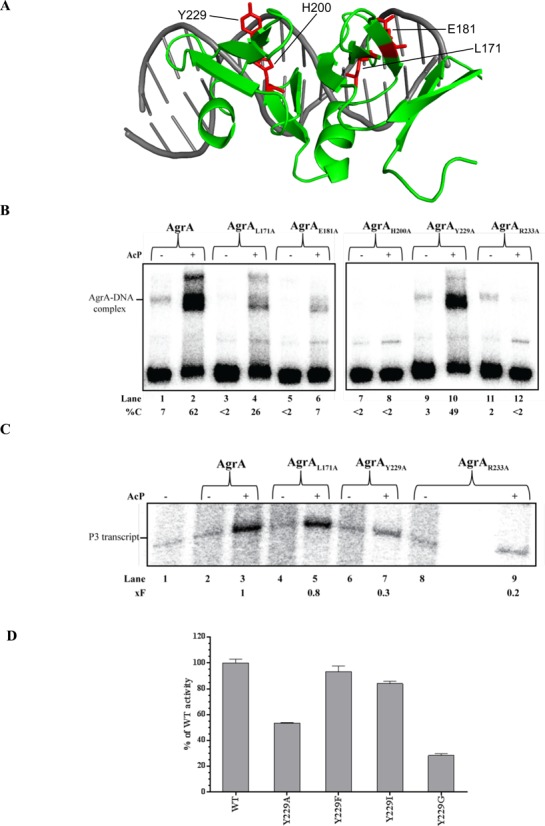
Conserved amino acid Y229 in the LytTR domain of AgrA is not required for DNA binding or bending but for transcription activation. (**A**) As in Figure 1B with the residues, which when substituted with alanine confer the putative TAD phenotype indicated in red. (**B**) Autoradiograph image of a 4–20% (w/v) native polyacrylamide gel comparing the ability of the four TAD mutants AgrA_L171A_ (lanes 3, 4), AgrA_E181A_ (lanes 5, 6), AgrA_H200A_ (lanes 7, 8), AgrA_Y229A_ (lanes 9, 10) with that of wild-type AgrA (lanes 1, 2) to bind to a DNA probe representing the intergenic region of the *agr* operon (as shown in Figure 1A) in absence (lanes 1, 3, 5, 7, 9, 11) and presence (lanes 2, 4, 6, 8, 10, 12) of acetyl phosphate. Lanes 11 and 12 contain the DNA-binding mutant AgrA_R233A_, which is used here as a negative control. The percentage of DNA bound by AgrA (%C) in each reaction is given at the bottom of the gel. (**C**) Autoradiograph image of a 10% (v/v) denaturing urea gel showing the synthesis of the P3 transcript by wild-type AgrA (lanes 2, 3), AgrA_L171A_ (lanes 4, 5), AgrA_Y229A_ (lanes 6, 7) and AgrA_R233A_ (lanes 8, 9) in the absence (lanes 2, 4, 6, 8) and presence (lanes 3, 5, 7, 9) of acetyl phosphate. The fold decrease in transcription (xF) compared to the P3 transcript in the presence of activated AgrA (lane 3) in each reaction is given at the bottom of the gel. (**D**) Graph showing GFP expression [as GFP fluorescence units (GFP-FU)] as a function of growth (OD600) relative to the SH1000^−^_agr IR P3-GFP_ + pSN-P2-*agrA* strain (WT) for SH1000^−^_agr IR P3-GFP_ containing pSN-P2-*agrA_Y229A_*, pSN-P2-*agrA_Y229F_*, pSN-P2-*agrA_Y229I_* and pSN-P2-*agrA_Y229G_* after 8 h of growth in TSB. For (B) and (C) at least two independent experiments were done to obtain the %C and xF values, respectively. The data obtained were all within 5–10% of the values shown. Data for (D) were obtained from at least three biological replicates.

In the crystal structure of the AgrA LytTR domain-DNA complex, aa residues L171, E181, H200 and Y229 are located proximal to the DNA (Figure 3A). We initially wanted to determine, using purified proteins, if alanine substitution at L171, E181, H200 and Y229 impairs the DNA binding activity of the mutant AgrA protein, and thereby contributes to the observed TAD phenotype. To test the DNA-binding activity of the L171A, E181A, H200A and Y229A AgrA mutants, we conducted electrophoretic mobility shift based protein-DNA-binding assays with a 214 bp long DNA probe containing the intergenic region of the *agr* operon and with the purified AgrA mutants. Since AgrA requires phosphorylation by AgrC for specific binding to DNA (see above), *in vitro* phosphorylation of AgrA was achieved using the small phosphodonor acetyl phosphate. The AgrA_R233A_ mutant served as the negative control in the protein-DNA-binding assays. As shown in Figure 3B, and as expected, phosphorylation with acetyl phosphate markedly increased the binding of wild-type AgrA to the DNA probe (compare lanes 1 and 2) and AgrA_R233A_ mutant did not detectably bind to the DNA probe in the presence of acetyl phosphate compared to wild-type AgrA (compare lanes 11 and 12), thus indicating the specific binding of AgrA to the DNA probe in the presence of acetyl phosphate. Of the four putative TAD mutants, AgrA_Y229A_ bound to the DNA probe to a level comparable to the wild-type AgrA, whilst AgrA_L171A_, AgrA_E181A_ and AgrA_H200A_ bound to the DNA probe with different degrees of reduced efficiency compared to wild-type AgrA (Figure 3B, lanes 3–10). Overall, the results indicate that an alanine substitution at aa residue Y229 in the LytTR domain of AgrA, whilst only moderately affecting DNA-binding, significantly impairs the ability of AgrA to activate transcription. Further, since the AgrA_Y229A_ mutant binds DNA in a phosphorylation-dependent manner, we can exclude the possibility that, under the assay conditions, the TAD property of this mutant is due to defects associated with phosphorylation.

To ascertain that AgrA_Y229A_ is a *bona fide* TAD mutant, we tested the ability of the AgrA_Y229A_ to activate transcription from the *agr* operon P3 promoter *in vitro*. The AgrA_L171A_ mutant was also included in the *in vitro* transcription assays as it displayed the best DNA binding activity (∼50% wild-type activity; Figure 3B, compare lanes 2 and 4) compared to AgrA_E181A_ and AgrA_H200A_ mutants. As shown in Figure 3C, phosphorylated AgrA_Y229A_ failed to activate transcription from the P3 promoter to a level comparable to that seen in reactions containing wild-type AgrA (compare lanes 2 and 3 with lanes 6 and 7). In contrast, phosphorylated AgrA_L171A_ activated transcription from the P3 promoter to a level almost comparable to that of wild-type AgrA (compare lanes 2 and 3 with lanes 4 and 5), even though this mutant binds DNA approximately 50–60% less efficiently than the AgrA_Y229A_ mutant or wild-type AgrA.

Tyrosine is a polar, hydrophobic and aromatic aa residue. To investigate the role of the tyrosine residue at position Y229 we made the AgrA_Y229F_, AgrA_Y229I_ and AgrA_Y229G_ mutants and compared their ability to activate transcription from the P3 promoter *in vivo*. As shown in Figure 3D, substitution of Y229 by the non-polar, hydrophobic and aromatic aa phenylalanine did not significantly affect the ability of the AgrA_Y229F_ mutant to activate transcription. Similarly, substitution of Y229 by the non-polar, hydrophobic, and non-aromatic aa isoleucine, which contains a bulky side chain as tyrosine did not significantly affect the ability of the AgrA_Y229I_ to activate transcription. In contrast, substitution of Y229 by the small non-polar, hydrophobic, non-aromatic aa glycine significantly impaired the ability of AgrA_Y229G_ mutant to activate transcription, as seen with AgrA_Y229A_. Overall, the results suggest that the bulky hydrophobic side chain of tyrosine at position 229 in the LytTR domain in AgrA is required for maximal activation of transcription.

**Figure 4.**
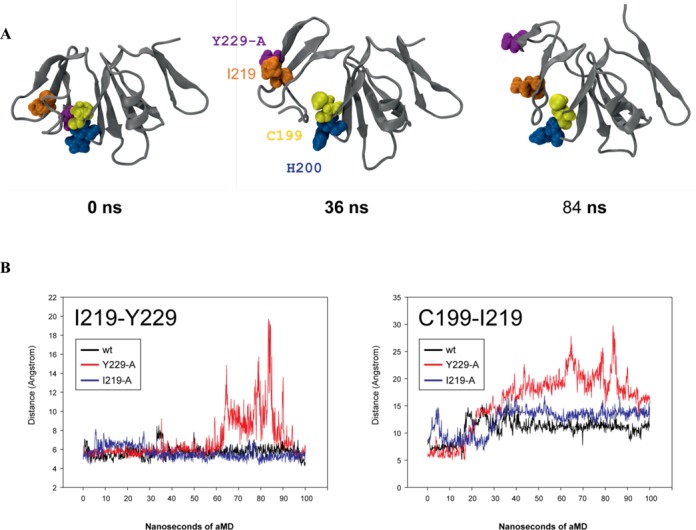
Molecular dynamics analysis of AgrA LytTR domain. (**A**) Snapshots of the aMD simulation of Y229-A at different time points. The polypeptide chain of the AgrA LytTR domain is shown as a grey ribbon. The side chains of the aas of interest are represented as van der Waals spheres (C199 yellow, H200 blue, I219 orange and the Y229-A *in silico* substitution in purple). At the beginning of the simulation (t = 0 ns), Y229-A is in close contact (as Y229 would be in the wild-type structure) with C199 and H200. This association dissociates during the course of the simulation, resulting in the carboxyl-terminal three β-sheets dissociating from the remainder of the LytTR domain (t = 36). At a later stage, Y229-A and I219 dissociate from each other (t = 84). (**B**) Quantitation of the aMD results shown in (A): The center-of-mass distances between I219 and Y229-A (left panel) and I219-A (right panel) are plotted against aMD simulation time for the wild-type (wt) structure (black), Y229-A (red) and I219-A (blue). The distance between I219 and Y229-A increases substantially at one stage of the simulation (from 55 ns onwards), but appears to reform in the final stages of the simulation. In contrast, the wild-type and I219-A simulations maintain a high degree of stability of this interaction. The distance between C199 and I219 serves as a measure of the dissociation of the three carboxyl-terminal β-sheets.

To further interrogate the interactions made by aa residue Y229 in AgrA, we carried out a series of fully atomistic accelerated molecular dynamics (aMD) stimulations. aMD is a powerful technique to explore the conformational space available to macromolecules (30) and is thus a particularly effective method for investigating structural changes induced by mutations. Simulation of the wild-type AgrA LytTR domain revealed that the core of the structure is very stable and deviates only within the expected range (root-mean square deviation < 2.4 Å) from the original crystal structure during 100 ns of aMD simulation. In contrast, simulation of AgrA LytTR domain containing the *in silico* Y229-A substitution under identical conditions shows a substantial destabilization of the carboxyl-terminal domain (Figure 4; Supplementary movie 1). In the wild-type structure Y229 makes close contact with C199, H200 and I219 based on a dense network of van der Waals contacts, hydrogen-bonding and hydrophobic interactions. These contacts are severely disrupted in Y229-A, resulting in the rapid dissociation of the three C-terminal β-strands from the main β-sheet transversing the LytTR domain. At a later stage of the simulation, it also becomes evident that Y229-A further destabilizes the two carboxyl-terminal β strands and causes a reversible unfolding of this sub-structure due to the absence of interaction between I219 and Y229. Taking into account the structural relevance of the interaction between I219 and Y229, it appeared initially puzzling that the I219A substitution displayed no discernible defect in activating transcription from the P3 promoter (Figure 2A). Simulations of the *in silico* I219-A substitution revealed, however, that in this situation Y229 forms an alternative interaction network involving the adjacent residues E217 and R218 (Figure 4; Supplementary movie 2). It is therefore evident, from both theoretical as well as experimental observations, that I219 plays a structurally redundant role (which also explains the evolutionary variability in this position (Supplementary Figure S1)). Overall the aMD simulations show that an alanine substitution at Y229 is predicted to have a substantial impact on the carboxyl-terminal part of the LytTR domain of AgrA. The mutation causes a ‘localized’ structural destabilization that may prevent AgrA from adopting a conformation required for transcription activation, while still retaining DNA-binding activity.

**Figure 5.**
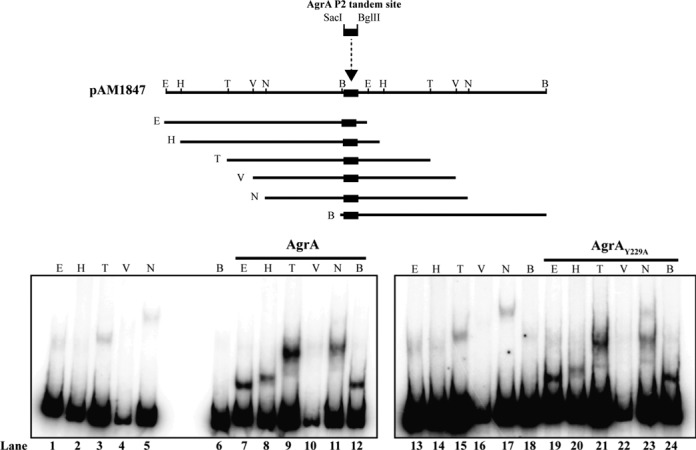
A schematic (based on Reyes *et al*. (14)) of the DNA bending vector pAM1847 containing the AgrA P2 tandem binding site (represented by a black rectangle) cloned into the *Sac*I-*BglI*I site. The *EcoR*I-*Sac*I fragment on the 5′ side of the AgrA P2 binding site is identical to the *BglI*I-*BamH*I fragment on the 3′ side of the AgrA P2 binding site. Hence, digestion of the recombinant vector with any of the six restriction enzymes *EcoR*I (E), *Hind*III (H), *BstN*I (N), *EcoR*V (V), *Nhe*I (N) or *BamH*I (B) produces DNA fragments of identical length but with a different position of the AgrA P2 binding site with respect to the 5′ and 3′ end of the fragment. The autoradiograph image of a 4.5% (v/v) nondenaturing polyacrylamide gel shows the mobilities of phosphorylated AgrA_Y229A_ and wild-type AgrA-DNA complexes bound to each of the DNA fragments generated upon digestion of pAM1847 with *EcoR*I (E), *Hind*III (H), *BstN*I (N), *EcoR*V (V), *Nhe*I (N) or *BamH*I (B). Lanes 1–6 and 13–18 contain no protein. Data from at least two independent experiments.

A previous study by Reyes *et al*. reported that AgrA induced bending of DNA is the main driving force for activation of transcription from the P2 and P3 promoters (14). However, based on the results above (Figures 3D and 4), it is unlikely that Y229 is involved in the DNA bending activity of AgrA. Therefore, to directly rule out this possibility, we carried out the DNA bending assay previously used by Reyes *et al*. to compare the DNA bending activity of AgrA_Y229A_ mutant with that of wild-type AgrA in the presence of acetyl phosphate. This assay uses the pAM1847 plasmid containing the AgrA tandem binding site upstream of the P2 promoter region cloned between the *Sac*I and *Bgl*I sites (schematic in Figure 5). Digestion of the plasmid with *EcoR*I, *Hind*III, *BstN*I, *EcoR*V, *Nhe*I and *BamH*I results in DNA fragments of identical length and composition in which only the position of the AgrA binding sites is permuted with respect to the 5′ terminus of the fragments (schematic in Figure 5). Therefore, AgrA-mediated DNA bending can be monitored by nondenaturing polyacrylamide electrophoretic analysis of the AgrA–DNA complexes, as the mobility of these will be strongly dependent on the position of the bend in the DNA molecule as a result of AgrA binding. As shown in Figure 5, no detectable differences were seen in the pattern of the mobilities of the wild-type and mutant AgrA–DNA complexes, thus indicating that AgrA_Y229A_ and wild-type AgrA bend the DNA equally well and that conserved aa Y229 in the LytTR domain of AgrA is not a major determinant of the DNA bending activity of AgrA.

## DISCUSSION

Our analysis has provided several novel insights into AgrA function in *S. aureus* and other LytTR containing transcription factors:

The *agr* operon indirectly controls the expression of hemolysins in *S. aureus* via RNAIII and directly via AgrA (8). Lack of hemolytic activity is typical of strains with dysfunctional *agr* operon. A dysfunctional *agr* operon is considered to provide an adaptive advantage for survival in the infected host but counter-adaptive outside infected host tissues (31). Under laboratory growth conditions, spontaneous mutations in the *agr* operon genes can occur, which can confer a nonhemolytic phenotype and this has also been observed for the widely used laboratory strain SH1000, with the nonhemolytic variant designated SH1000^−^ (29). Our results reveal that the molecular basis for the *agr* operon dysfunction in SH1000^−^ is a point mutation at a conserved aa residue (H174) in AgrA, which disrupts salt–bridge interactions that stabilizes the interaction between three β sheets in the LytTR domain of AgrA. Further, a phenylalanine substitution at aa residues L171 in AgrA has been previously reported to confer the nonhemolytic phenotype (indicative of *agr* operon dysfunction) in a nosocomial methicillin-resistant *S. aureus* isolate (31). Intriguingly, our results reveal that even though AgrA_L171A_ mutation displays a reduced (by ∼50%) DNA binding activity compared to wild-type AgrA, its ability to activate transcription is only moderately affected (∼20% reduction compared to wild-type AgrA). Thus, it is possible that an aromatic side chain at position 171 in the LytTR domain is more deleterious for AgrA activity than the presence of alanine.

The results reveal that alanine substitutions at aa residues L171, E181 or H200 significantly impair the DNA-binding activity of AgrA. It is possible that alanine substitutions at these aa residues indirectly affect the ability of mutant AgrA to become phosphorylated, however, this is unlikely given their proximity to DNA in the context of the structure of the AgrA LytTR domain–DNA complex (Figure 3A). The most conserved sequence motif within the LytTR domain is FhRhHRS (where ‘h’ indicates a hydrophobic aa) (3). In AgrA, the FhRhHRS corresponds to FFRCHNS (residues 196–202). In the crystal structure of the AgrA LytTR domain–DNA complex only aa residues R198, N201 and S202 were shown to be involved in interaction with DNA and alanine substitutions at N201 reduced the ability of the AgrA LytTR domain to bind DNA (4). These observations clearly imply a role for aa residue H200 within the highly conserved FFRCHNS motif in the binding of AgrA to DNA, and, based on the proximity of H200 to DNA in the AgrA LytTR domain–DNA complex, base-specific contacts between H200 and DNA cannot be excluded. In further support of this view, alanine substitution of H188 (equivalent residue of H200 in AgrA) in the LytTR domain of *Clostridium perfringens* RR-TF VirR, which regulates virulence and toxin gene expression, confers a loss of activity phenotype and residues R186 and S190 in the FhRhHRS motif of VirR has been shown to be involved in DNA-binding (5).

Importantly, the results identify the highly conserved aa residue Y229 in the LytTR domain of AgrA as a key determinant for maximal transcription activation by AgrA and the AgrA_Y229A_ mutant still retains some level of activity (40–50% *in vivo* and 30% *in vitro* compared to wild-type AgrA) to activate transcription. In the structure of the AgrA LytTR domain–DNA complex, Y229 is adjacent to an aa residue critical for AgrA folding (C228) and DNA interaction (R218), thus indicating that Y229 is part of a multifunctional region in AgrA. Consistent with this notion, a recent study reported that a novel antibacterial compound, called savarin, binds to the LytTR domain of AgrA in a region proximal to aa residues C228, R218 and Y229 and thereby abrogates AgrA function (32). The proximity of both AgrA-binding sites to the P2 and P3 core promoter elements (Figure 1A) suggests that transcription activation at both promoters could involve direct protein–protein interaction between the region containing Y229 in AgrA and the RNAp and thus could occur via a simple ‘recruitment’ mechanism, whereby AgrA could facilitate the binding of RNAp to the promoter to yield a transcriptionally proficient promoter complex. Furthermore, a previous study by Reyes *et al*. reported that transcription from P2 and P3 agr operon promoters is differentially regulated, with the former (P2) dependent on AgrA and SarA and the later dependent only on AgrA for maximum promoter activity (14). Therefore, it is possible that the region containing Y229 in AgrA is involved in interaction with SarA for the activation and that the SH1000^−^_agr IR P3-GFP_ reporter strain used in this study indirectly indicates P2 activity (which drives its own transcriptio, see Materials and Methods). However, this is unlikely because AgrA_Y229A_ mutant displays the same level of activity in the context of pSN-P2-*agrA* and pSN-*itet*-*agrA* in SH1000^−^_agr IR P3-GFP_ strain. The aMD analysis reveals that an alanine substitution at Y229 could cause a ‘localized’ structural destabilization that may prevent AgrA from adopting a conformation required for efficient transcription activation. This observation further substantiates the multifaceted role of Y229 in transcription activation and maintaining local structural integrity of AgrA. Future work will focus on molecular details of such potential interactions in order to further delineate the mechanism by which AgrA activates transcription.

## SUPPLEMENTARY DATA

Supplementary Data are available at NAR Online.

SUPPLEMENTARY DATA
