# Supplementary material for: Systematic mutational analysis of the LytTR DNA binding domain of Staphylococcus aureus virulence gene transcription factor AgrA
Source: Nucleic Acids Res. 2014 Oct 28;42(20):12523–36. doi: 10.1093/nar/gku1015 (PMC4227749; doi:10.1093/nar/gku1015)
Supplement: SUPPLEMENTARY DATA [file supp_42_20_12523__index.html]

Systematic mutational analysis of the LytTR DNA binding domain of Staphylococcus aureus virulence gene transcription factor AgrA — Systematic mutational analysis of the LytTR DNA binding domain of Staphylococcus aureus virulence gene transcription factor AgrA — SUPPLEMENTARY DATA 

# Systematic mutational analysis of the LytTR DNA binding domain of *Staphylococcus aureus* virulence gene transcription factor AgrA

## SUPPLEMENTARY DATA

**Files in this Data Supplement:**

- SUPPLEMENTARY DATA
- SUPPLEMENTARY DATA
- SUPPLEMENTARY DATA
