# Supplementary material for: Systematic mutational analysis of the LytTR DNA binding domain of Staphylococcus aureus virulence gene transcription factor AgrA
Source: Nucleic Acids Res. 2014 Oct 28;42(20):12523–36. doi: 10.1093/nar/gku1015 (PMC4227749; doi:10.1093/nar/gku1015)
Supplement: SUPPLEMENTARY DATA [file supp_gku1015_nar-02690-m-2014-File003.pdf]

Systematic mutational analysis of the LytTR DNA binding domain of *Staphylococcus aureus* virulence gene transcription factor AgrA.

Nicod et al.

SUPPLEMENTARY FIGURE LEGENDS

Fig. S1. Multiple sequence alignment of the AgrA LytTR domain of representative staphylococci strains. Conserved residues are represented by a dot. The aa residues displaying similar colours have similar properties. The conserved histidine at position 174 is highlighted in yellow.

Fig. S2. Image of a Western blot showing accumulation of AgrA in whole-cell lysates of SH1000$^{-}_{\text{agr IR P3-GFP}}$ + pSN-*itet-agrA* over time grown in rich media. The intensity of the band corresponding to AgrA is quantified and expressed in arbitrary units.

SUPPLEMENTARY TABLES

Table S1. Bacterial strains and plasmids used in this study.

Table S2. Primers used for cloning and EMSA.

SUPPLEMENTARY MOVIES

Supplementary movie 1. aMD simulations showing the consequences of the *in silico* Y229-A mutation on AgrA LytTR domain conformation (see text for details). The Y229-A, I219, C199 and H200 aa residues are shown as van der Waals spheres in purple, orange, yellow and blue, respectively.

Supplementary movie 2. aMD simulations showing the consequences of the *in silico* I229-A

mutation on AgrA LytTR domain conformation (see text for details). The Y229, I219-A, E217 and R218 aa residues are shown as van der Waals spheres in dark green, purple, red and blue, respectively.

Nicod et al

Fig. S1

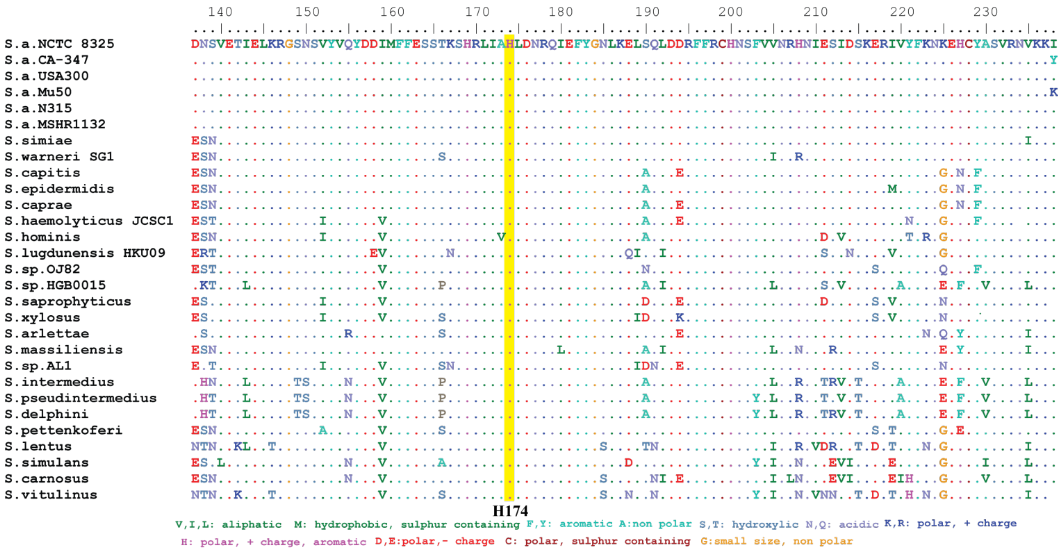

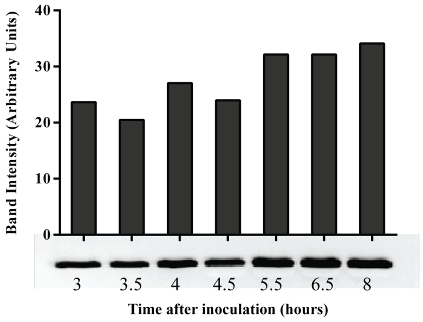
40
30
20
10
0
Band Intensity (Arbitrary Units)
3
3.5
4
4.5
5.5
6.5
8
Time after inoculation (hours)

Table S1. Bacterial strains and plasmid used in this study

Strain/plasmid	Genotype/characteristics*	Source/reference
Strains		
E. coli		
XL1-blue	Efficient cloning strain	Stratagene
DC10B	Δ*dcm* in the DH10B background; Dam methylation only	Monk et al. (2012)
ER2566	Expression strain, IPTG inducible T7 RNA polymerase<br>Deficient in *lon* and *ompT* proteases	New England Biolabs
BL21(DE3)	Expression strain, IPTG inducible T7 RNA polymerase	Stratagene
S. aureus		
SH1000	Functional *rbsU* derivative of NCTC8325-4 *rbsU*	Horsburgh et al. (2002)
SH1000-	*agr*-deficient SH1000 because of H174L mutation in a*grA*<br>SH1000	Tsompanidou et al. (2010), this study
SH1001	a*gr::tet*	Horsburgh et al. (2002)
SH1000-$_{agr\ IR\ P3\text{-}GFP}$	pCL55$_{agr\ IR\ P3\text{-}GFP}$ integrated into<br>SH1000-	this study
SH1000 $_{agr\ IR\ P3\text{-}GFP}$	pCL55$_{agr\ IR\ P3\text{-}GFP}$ integrated into SH1000	James et al. (2013)
SH1001 $_{agr\ IR\ P3\text{-}GFP}$	pCL55$_{agr\ IR\ P3\text{-}GFP}$ integrated into SH1001	James et al. (2013)
Plasmids		
pCN34	Shuttle vector for Gram-positive bacteria.<br>Amp^R in *E. coli*, Kan^R in *S. aureus*	Chapentier et al. (2004)
pCN34*itet*	pCN34 carrying a tetracycline inducible promoter<br>Amp^R in *E. coli*, Kan^R in *S. aureus*	Corrigan et al. (2011)
pCN44	Shuttle vector for Gram-positive bacteria.<br>Amp^R in *E. coli*, Erm^R in *S. aureus*	Chapentier et al. (2004)
pCL55 $_{agr\ IR\ P3\text{-}GFP}$	pCL55 carrying P2GFP	James et al. (2013)
pTYB2	Expression vector carrying a self-cleavable affinity tag<br>Amp^R	New England Biolabs
pJR$_{P2+P3}$	Promoter template for *in vitro* transcription<br>Amp^R	Reynolds and Wigneshweraraj (2011)
pJR28-[6His]*rpoD*	Expression vector carrying *rpoD*<br>Kan^R	Reynolds and Wigneshweraraj (2011)
pAM1847	DNA bending vector, pCY7 containing the AgrA P2 tandem site<br>Amp^R	Reyes et al. (2011)
pSN-P2-*agrA*	pCN34 carrying *agr* IR P2-*agrA*	this study
pSN-*itet-agrA*	pCN34*itet* carrying *agrA*	this study
pSN-*agrA*	pTYB2 carrying *agrA*	this study

* Amp, ampicillin; Cm, chloramphenicol; Kan, kanamycin; Erm, erythromycin.

Table S2. Primers used for cloning and EMSA

Primer	Use	Sequence (5'-3')
P2-RBS F (for P2-AgrA)	cloning P2-RBS-AgrA-TT into pCN34; fuse to AgrA. *BamH*I site.	CGCGGATCCCCGGAATTCTTTTTCTTAACTAGTCG
P2-RBS R (for P2-AgrA)	cloning P2-RBS-AgrA-TT into pCN34; fuse to AgrA. Contains RBS.	CGCAAATGAAAATTTTCATTTTTTTTTCCTCCTTAGGATCACC
P2-AgrA F (for P2-AgrA)	cloning P2-RBS-AgrA-TT into pCN34; fuse to P2-RBS	ATGAAAATTTTCATTTGCGAAGACGATCCAAAAC
P2-AgrA R (for P2-AgrA)	cloning P2-RBS-AgrA-TT into pCN34; fuse to P2-RBS	GCATTTAGAATATTATATTTTTTTAACGTTTCTCACCG
P2-TT F (for P2-AgrA)	cloning P2-RBS-AgrA-TT into pCN34; fuse toP2-RBS-AgrA.	CGTTAAAAAAATATAATATTCTAAATGCATAATAAATACTG
P2-TT R (for P2-AgrA)	cloning P2-RBS-AgrA-TT into pCN34itet; *Kpn*I site.	CGGGGTACCTGTCACTTTGCTTGATATATGAGAATTATTTAACC
AgrA pCN34*itet* F	cloning RBS-AgrA-TT into pCN34. Contains RBS. *KpN*I site.	CGGGGTACCGATCCTAAGGAGGAAA
AgrA pCN34*itet* R	cloning RBS-AgrA-TT into pCN34. *EcoR*I site.	CCGGAATTCTGTCACTTTGCTTGATATATGAGAATTATTTAACC
AgrA pTYB2 F	cloning AgrA into pTYB2. *Nde*I site.	GAAGCATATGAAAATTTTCATTTGCGAAGACG
AgrA pTYB2 R	cloning AgrA into pTYB2. *Sma*I site.	GAAGCCCGGGTATTTTTTTAACGTTTCTCACCGATGC
P2-IR-P3 F	EMSA probe	ACCACTCTCCTCACTGTCATTATACG
P2-IR-P3 R	EMSA probe	ACATCTCTGTGATCTAGTTATATTAAAACATGC
